# Assessing the feasibility of integration of self-care for filarial lymphoedema into existing community leprosy self-help groups in Nepal

**DOI:** 10.1186/s12889-018-5099-0

**Published:** 2018-01-30

**Authors:** Joseph Pryce, Hayley E. Mableson, Ramesh Choudhary, Basu Dev Pandey, Dambar Aley, Hannah Betts, Charles D. Mackenzie, Louise A. Kelly-Hope, Hugh Cross

**Affiliations:** 1 Centre for Neglected Tropical Diseases, Department of Parasitology, Liverpool School of Tropical Medicine, Liverpool, UK; 2 Nepal Leprosy Trust, Kathmandu, Nepal; 3 Department of Health Services, Kathmandu, Nepal; 4 American Leprosy Missions, Greenville, USA

**Keywords:** Nepal, Lymphatic filariasis, LF, Elephantiasis, Leprosy, *Wuchereria bancrofti*, *Mycobacterium leprae*, Self-help group, Stigma, Disability

## Abstract

**Background:**

Lymphatic filariasis (LF) and leprosy are disabling infectious diseases endemic in Nepal. LF infection can lead to lymphoedema and hydrocoele, while secondary effects of leprosy infection include impairments to hands, eyes and feet. The disabling effects of both conditions can be managed through self-care and the supportive effects of self-help groups (SHGs). A network of SHGs exists for people affected by leprosy in four districts in Nepal’s Central Development Region, however no such service exists for people affected by LF. The aim of this study was to determine the feasibility of integrating LF affected people into existing leprosy SHGs in this area.

**Methods:**

A survey was conducted using a semi-structured questionnaire to elicit information on: (i) participant characteristics, clinical manifestation and disease burden; (ii) participants’ knowledge of management of their condition and access to services; and (iii) participants’ knowledge and perceptions of the alternate condition (LF affected participants’ knowledge of leprosy and vice versa) and attitudes towards integration.

**Results:**

A total of 52 LF affected and 53 leprosy affected participants were interviewed from 14 SHGs. On average, leprosy affected participants were shown to have 1.8 times greater knowledge of self-care techniques, and practiced 2.5 times more frequently than LF affected participants. Only a quarter of LF affected participants had accessed a health service for their condition, compared with 94.3% of leprosy affected people accessing a service (including SHGs), at least once a week. High levels of stigma were perceived by both groups towards the alternate condition, however, the majority of LF (79%) and leprosy (94.3%) affected participants stated that they would consider attending an integrated SHG.

**Conclusions:**

LF affected participants need to increase their knowledge of self-care and access to health services. Despite stigma being a potential barrier, attitudes towards integration were positive, suggesting that the SHGs may be a good platform for LF affected people to start self-care in this area.

**Trial registration:**

This is not a registered trial.

## Background

Lymphatic filariasis (LF) is a Neglected Tropical Disease (NTD) that causes significant disability worldwide, with an estimated 36 million people suffering from chronic complications of the disease [1]. LF is primarily caused by the parasite *Wuchereria bancrofti,* which is transmitted to humans through the bite of an infected mosquito. Infection is commonly acquired during childhood, initially causing unseen damage to the lymphatic vessels. Disfiguring and stigmatising symptoms such as lymphoedema and hydrocoele can transpire later in life. Lymphoedema is most commonly a painful swelling of the limbs that results from persistent aggravation of the lymphatic vessels and consequent lymphatic incompetence. The condition, which has both acute and chronic phases, may affect a person’s capacity to walk, stand or sit, as well as their ability to carry out activities of daily living [2, 3]. Hydrocoele is an intra-scrotal swelling that has also been shown to impact activities of daily living, and as a consequence affects men’s economic activity, productivity and relationships [4–7].

Leprosy is also a NTD associated with impairment, activity limitation and marginalisation. It is caused by *Mycobacterium leprae* bacteria, and infection in its early stages is characterised by hypopigmented patches in the skin with loss of sensation. The long-term physical impairments can be wide-ranging, particularly if treatment is delayed. *M. leprae* targets Schwann cells, with the resultant neuropathy leading to a possible loss of sensory, motor and autonomic function. Secondary effects of peripheral neuropathy can include visible impairments to hands, eyes and feet, depending on which nerves are affected. In many countries where leprosy is endemic, it is the visibility of secondary effects such as painless wounds, lagophthalmos and foot drop that are stigmatising [8]. In the last 30 years, control of leprosy has improved significantly, particularly through the combined efforts of the WHO and non-profit organisations to provide Multi-Drug Therapy (MDT) to all infected people. Over 15 million people were reported to be cured of the disease between 1985 and 2010 [9]. The number of new cases detected annually is showing a decline, falling from 407,791 globally in 2004, to 210,758 in 2014 [10, 11].

Beyond the physical challenges that each disease is associated with, the effects of stigma can be profound. Men affected by LF face challenges in establishing relationships and in securing financial stability, with reports that some patients having been prohibited from trading their produce at local markets [4, 12]. Multiple studies have documented the limited marriage prospects of women with lymphoedema; explanations given were that these women fail to meet the aesthetic standards held by society, and that their ability to assist in harvesting home-grown produce is restricted [12, 13]. A study in East Nepal found the behaviours of rejecting and ostracising a wife affected by leprosy, or taking a second wife as a replacement, were common [14]. The psychological stresses of these exclusions can be severe, particularly in South Asian countries, where to be excluded from a family or community is to be deprived of any sense of purposeful function in life.

Regular self-care can prevent worsening of lymphoedema [15]. The WHO-recommended basic lymphoedema management activities include limb washing, elevation, exercise, skincare, wound-care (applying creams and dressings) and the protection of feet with appropriate footwear [16]. These practices can be taught through community groups and can be performed by affected people at home. The emphasis on home-based self-care and simple community-level intervention resonates with the WHO’s disability prevention guidelines for leprosy; these suggest that while healthcare workers may be the primary source of disability prevention advice, self-care with guidance from peers similarly affected by leprosy can be extremely beneficial [17]. The commonalities between the basic interventions for LF and leprosy include daily home-based self-care, procedures for which include the following: avoidance of injury; skin and wound care; prevention of contractures; compliance with footwear advice and interventions to prevent activity limitation. Other morbidity management interventions that may be needed for both groups include advice and support for caregivers; surgery to address impairments; schemes for economic upliftment; advocacy and social mobilisation [15].

**Fig. 1:**
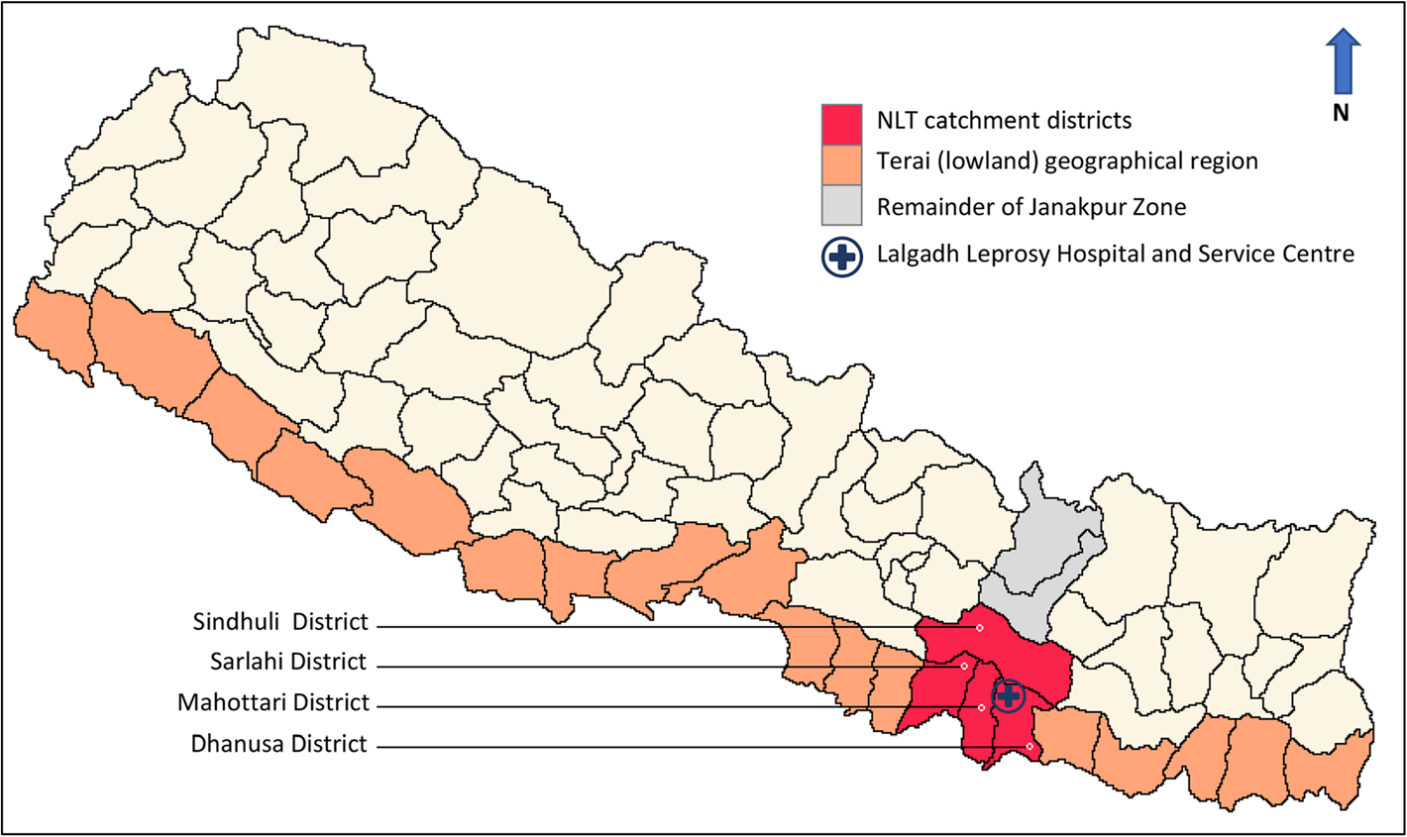
Map of Nepal and the study area in the Terai region of Janakpur Zone

The supportive and motivational effects of attending community-level self-help groups (SHGs) are promoted for leprosy and LF alike [15, 18]. The benefits of SHGs for leprosy are well documented, and participation in SHGs has been shown to improve patients’ lives with respect to both disability prevention and social participation [19, 20]. In Nepal, there are several organisations that facilitate SHG development. The Nepal Leprosy Trust (NLT) facilitates 101 leprosy SHGs in four districts in the Janakpur Zone of the Central Development Region of Nepal (Fig. 1). Through a range of activities including disability management, micro-enterprise, initiating and implementing community development projects, the SHGs have been shown to have gained community respect, which has increased opportunities for participation in community activities. NLT’s empowerment approach has led to a decrease in community stigma [20, 21].

Community-based care and morbidity management interventions have been shown to have similar benefits for those affected by filarial lymphoedema [22–24]. The Ministry of Health, Nepal had only recently begun to scale up Morbidity Management and Disability Prevention (MMDP) activities as part of its National LF elimination strategy, therefore there were no such community-based programmes for people affected by filarial lymphoedema in the south of the Central Development Region. This provided an opportunity to provide training and care to people affected by LF by integrating lymphoedema management with the leprosy self-care programme implemented by NLT.

The WHO LF MMDP guidelines do suggest the integration of MMDP services with those of other disabling diseases, such as leprosy [15]. However, there is currently little evidence to guide how these integrated services can be practically implemented. The aim of this study, therefore, was to determine the feasibility of integrating people affected by filarial lymphoedema into SHGs for people affected by leprosy, in the catchment districts of NLT in the Central Development Region of Nepal. The study had two main objectives: to determine whether there was a need for integration, and to evaluate the perspectives of people affected by each disease on the feasibility of an integrated service. The authors sought to develop an understanding of the affected people’s knowledge of the alternate disease; their perception of stigma towards the disease in their community, and their willingness to participate in an integrated service.

## Methods

### Study site

The NLT Lalgadh Leprosy Hospital and Services Centre (LLHSC), located in the Terai (lowlands) of the Janakpur Zone of the Central Development Region, Nepal (Fig. 1), provides general health services in addition to services for leprosy affected people. The hospital also oversees 101 established SHGs for people affected by leprosy in the four districts (Dhanusa, Mahottari, Sindhuli and Sarlahi) that surround the LLHSC. SHGs were distributed so that there was no more than one in any Village Development Committee (VDC) catchment area; VDCs at the time of the study were the most peripheral level of local government, however, it is important to note that in 2017 they were replaced with Gaupalika when the administrative divisions of Nepal changed. For this study, three of the four NLT catchment districts were selected because they were considered to be the most endemic for LF, with lymphoedema case estimates ranging from 50 to 350 according to the Ministry of Health unpublished reports, however these numbers are potentially underestimated. The districts included in the study were Dhanusa, Mahottari and Sarlahi, in which there were a total of 91 SHGs.

### Study design

A cross-sectional survey of people affected by filarial lymphoedema (hereon refered to as participants affected by LF) and people affected by leprosy was conducted using a semi-structured questionnaire. Questionnaires were translated into the local language (Maithili) and were conducted in an interview scenario using a local research assistant who read the question to the participant and recorded their answer, and aimed to avoid misinterpretation of questions by repeating the answer back to the participant. Surveys were completed using an electronic tablet and Open Data Kit (ODK) mobile survey software (https://opendatakit.org/).

The questionnaire was tailored to each participant group by changing the disease name and related information accordingly, and was structured in three parts to elicit information on demographics, knowledge of disease, access to care, and knowledge and community perceptions of the ‘alternate condition’ (i.e. participants affected by LF/ were asked about leprosy and participants affected by leprosy were asked about LF).

The first part of the questionnaire elicited participant characteristics, clinical manifestation and clinical effects, and included questions on;

- Demographics (age, gender, education, marital status, employment)
- Clinical manifestation (affected body parts, severity of disease)
- Clinical effects (length of time with condition, mobility (LF affected participants only), number and length of acute attacks (LF-affected participants only)

The second part of the questionnaire was related to participants’ knowledge of management of their condition and access to services, and included questions on;

- Knowledge and practice of management of condition (methods for prevention of worsening of condition, tailored for each condition)
- Availability and access to services (if participants had consulted a medical professional about their condition, they were asked which services were known to them; which were utilised; how regularly services were used; what barriers to service use had been experienced and if they had a desire to increase access to services). The survey was based on a survey described by Stanton et al. [25].

The third part of the questionnaire was designed to gather information on participants’ knowledge and perceptions of the alternate condition (LF affected participants’ knowledge of leprosy and leprosy affected participants’ knowledge of LF) and their attitudes towards integration. It included questions on:

- Knowledge of the alternate condition (knowledge of the disease, understanding of cause of disease, knowledge of management of disease, source of knowledge)
- Closest relationship to a person affected by the alternate condition
- Community perceptions of the alternate condition, based on the Explanatory Model Interview Catalogue - Community Stigma Scale (EMIC-CSS) tool [26]
- Attitudes towards integrated morbidity management with participants from the alternate group (would participants be willing to attend an integrated group; would participants be willing to perform basic preventative care measures together)

### Sampling and data analysis

A segmented sampling strategy was used, with the aim of recruiting four participants affected by LF and four participants affected by leprosy interviewed from each of five VDCs in each of the three districts (total 60 participants affected by LF and 60 participants affected by leprosy), allowing analysis that would detect statistically significant differences between the two disease groups with 80% power. This sampling approach was used as the number of people affected by LF or leprosy varied considerably, and it helped to obtain a geographical spread across the districts. Further, a pragmatic approach to VDC selection was used, coinciding study visits with scheduled SHG meetings. Leprosy affected participants were recruited at random from SHG members present at the group meeting. As no network of LF-affected people existed in the area, SHG facilitators were asked to invite four people affected by LF who they knew from the local VDC to attend the group meetings, and participate in the study visit.

Inclusion criteria for participants of the study consisted of:

- LF/leprosy affected people who had provided informed consent
- LF/leprosy affected people who had lived in the district for more than 5 years (this ensured all information gathered was informed and reflective of the study area).
- Confirmed cases of filarial lymphoedema (as informed by a clinically-trained member of the CDD) / leprosy affected person (registered member of the SHG).

Data was retrieved from the Open Data Kit (ODK) online database and interpreted using Microsoft Excel to tabulate and visualise findings. All statistical analyses were carried out using SPSS (Version 23). The mean number of methods known and practiced by each group was calculated with standard deviation (SD) and compared between groups using the Kruskal-Wallace non-parametric test. The EMIC-CSS survey to assess the community perceptions of the alternate condition consisted of twelve questions. Each question had four answer options: ‘yes’, ‘possibly’, ‘no’ and ‘don’t know’, with scores of 2,1,0,0 allocated respectively, and a total possible survey score of 24. The total scores for each participant were calculated, and the mean scores were compared between groups with a Student’s T-test. A *p* value < 0.05 was considered statistically significant. The questions regarding attitudes towards integration had possible answers of ‘yes’, ‘probably’, ‘probably not’ and ‘no’, with the percentage of participants responding ‘yes’ and ‘probably’ considered to be positive.

## Results

### Section1: Characteristics of participants

In total 52 participants affected by LF and 53 participants affected by leprosy were interviewed (total 105) due to difficulties in locating participants in some VDCs (Table 1). Of the 52 participants affected by LF, the mean age was 48.1 years (male 49.2; female 47.0). Nearly half of the participants affected by LF (24; 46.2%) were illiterate, and 28 (53.8%) were literate without having accessed any formal education. The most common source of support was through family, charity-based provision or house work, which supported 22 (42.3%) participants. The majority of participants were married (38; 73.1%), while 9 participants (17.3%) were widowed and the remaining 5 participants (9.6%) were single (Table 2).

**Table 1:** Participants per disease group, district and gender

Group		LF			Leprosy			Total
District		Dhanusa	Mahottari	Sarlahi	Dhanusa	Mahottari	Sarlahi	
Gender	female	4	12	11	2	5	6	40
	male	8	8	9	11	15	14	65
Total participants per district		12	20	20	13	20	20	
Total per group		52			53			105

Note. Cells represent numbers

**Table 2:** Participants’ characteristics

Group		LF (*n* = 52)	Leprosy (*n* = 53)
Mean age (years)	Male	49.2	55.9
	Female	47.0	46.5
	Total	48.1	53.6
Literacy	Illiterate	24	34
	Literate (no formal education)	8	10
	Literate (education)	20	9
Source of income	Grow own vegetables or own animals	10	23
	Small business	11	16
	Paid employment	7	8
	Home work	12	1
	Pension	2	1
	Family support	9	3
	Charitable donations	1	1
Marital status	Married	38	49
	Widowed	9	3
	Single	5	1

Note. Apart from the mean age groups, all cells represent numbers

Of the 53 participants affected by leprosy, the mean age was 53.6 years (male 55.9; female 46.5). A total of 34 participants were illiterate (64.2%), a further 10 (18.9%) were literate without having completed any formal education and 9 participants (17%) were literate and had completed primary level education. A higher number of participants affected by leprosy supported themselves financially; in total, 23 (43.4%) primarily grew their own vegetables or owned animals and 16 (30.2%) ran small businesses. The remaining 5 (9.4%) were primarily supported by their family or through charitable donations. The majority were married (49; 92.5%), while 3 (5.7%) were widowed and 1 participant (1.9%) was single (Table 2).

Of the participants affected by LF, a total of 51 participants (98.1%) had the condition in their leg, including 14 (26.9%) displaying bilateral lymphoedema. Two participants (3.8%) had lymphoedema in their arm, one of which also displayed lymphoedema of both legs. The mean number of years’ participants had been affected by their disease was 10.7 years. The mean distance LF-affected participants could walk was 3.3 km (range 0–9 km). A total of 51 (98.1%) participants reported that they had experienced acute attacks in the last 6 months, with a mean of 4.47 attacks (range 0–20) during this time.

A total of 16 (30.2%) participants affected by leprosy had only one body part affected by their condition, while 33 participants (62.33%) had two body parts affected and the remaining four participants (7.5%) had three body parts affected. The mean number of years’ the leprosy affected participants had been affected by their disease was 12.0 years (Table 3).

**Table 3:** The affected body parts by participant group

Group		LF (*n* = 52)	Leprosy (*n* = 53)
Body part affected	Arm	2	32
	Leg	51	39
	Trunk	–	18
	Forehead	–	2
	Eyes	–	3
Severity (LF only)	Mild	33	–
	Moderate	13	–
	severe	6	–
Years affected by disease	0–5	11	8
	6–10	22	19
	11–15	10	11
	16–20	5	12
	> 20	3	4

### Section 2: Knowledge, practice and access of care

#### Knowledge and practice of self-care

Whilst 48 participants affected by LF (92.3%) were aware that management of their condition was possible, knowledge of the four recommended methods to manage their condition was low, with 26 participants protecting their feet with appropriate footwear (50.0%), 21 cleaning their limbs (40.4%), 16 applying creams (30.8%), and eight raising limbs and exercising (15.4%) (Table 4). Only 14 participants (26.9%) were aware of at least three of the four recommended methods and only 11 (21.2%) practiced at least three methods.

**Table 4:** *Knowledge and practice of self-care techniques by* LF and leprosy affected participants

	Know about	Practice	Reason if not practiced
LF (*n* = 52)			
Self-care methods			
Protect feet^a^	26	24	2- It is not practical
Clean limbs^a^	21	17	3- it takes too long; 1- it is not practical
Apply creams^a^	16	16	
Raise limb and exercise^a^	8	8	
Other or local treatments^b^	11	–	
Breadth of knowledge and practice			
At least 2 of the 4 recommended methods	27	23	
At least 3 of the 4 recommended methods	14	11	
All 4 recommended methods	3	3	
Mean number of methods	1.81 (SD = 0.951)	1.25 (SD = 1.404)	
Leprosy (*n* = 53)			
Self-care methods			
Protect Feet^a^	52	52	
Soak limbs in clean water^a^	48	45	3- it takes too long
Physical Exercise^a^	48	48	
Check eyes in Mirror^a^	22	18	2- it takes too long; 2- need help
Other or local treatments^b^	3	–	
Breadth of knowledge and practice			
At least 2 of the 4 recommended methods	52	51	
At least 3 of the 4 recommended methods	46	45	
All 4 methods	19	13	
Mean number of methods	3.21 (SD = 0.717)	3.08 (SD = 0.703)	

^a^methods of self-care as recommended by WHO

^b^other or local treatments that are not recommended by WHO

All 53 participants affected by leprosy (100%) were aware that management could help their condition, with the majority stating several of the four recommended methods of management; 52 stated protecting their feet with appropriate footwear (98.1%), 48 soaking their limbs (90.6%), and 48 performing physical exercises (90.6%). The importance of checking eyes was less well known, with only 22 participants (41.5%) stating this method. Of the 53 participants, 46 (86.8%) were aware of at least three of these four methods and 45 (84.9%) practiced at least three methods.

The mean number of techniques known by participants affected by LF was 1.81 (SD = 0.951) and the mean number of techniques practiced was 1.25 (SD = 1.404). Conversely, the mean number of techniques known by those affected by leprosy was 3.21 (SD = 0.717) (1.8 times greater than participants affected by LF) and the mean number of techniques practiced was 3.08 (SD = 0.703) (2.5 times greater than participants affected by LF). Both knowledge and practice of the four major self-care techniques for each condition differed significantly between leprosy- and LF affected participants when compared using the Kruskal-Wallace test (*p* < 0.0001).

#### Access to services and barriers to accessing preferred services

Of the 52 participants affected by LF, 37 (71.2%) believed that some services were available to them to help them manage their condition; 16 (30.8%) were aware of self-care groups and 34 (65.4%) of hospital-based care (Table 5). Only one participant affected by LF (1.9%) accessed a service at least once a week. Of the remaining 51 participants, 49 stated that they would like to access services more regularly. Of the 26 participants affected by LF who stated they would like to access hospital-based care more often, 17 of them described the cost of treatment as the primary barrier to access (Table 6). Of the 20 participants who stated that they would like to access self-care groups, 10 participants also gave the cost of the activity as the primary barrier, with five citing the distance to an appropriate group or access to transport as their primary concern.

**Table 5:** Perception of availability of services to assist in the management of their condition

	LF (n = 52)	Leprosy (n = 53)
Which services are available to you to help you manage your condition?		
Self-care	16	53
Homecare	0	3
Hospital	34	28
Health-centre	1	7
How often do you access services to help manage your condition?		
Never	39	2
Less than monthly	1	1
Monthly	11	0
Weekly	1	23
Daily	0	27

**Table 6:** Preferred service and primary barrier to accessing this service in participants affected by LF that access services less than once a week (*n* = 49) and expressed a desire to access services more frequently

	Self-care based	Homecare based	Health Centre based	Hospital based
Access to transport	1	0	0	2
Need someone to accompany	2	0	0	3
Cost of service	10	1	2	17
Service too far away	4	0	0	3
Time constraint	1	0	0	1
Don’t know	2	0	0	0

All 53 participants affected by leprosy were aware that some services were available to them to help them manage their condition. All leprosy affected participants were aware of self-care groups and 28 (52.8%) were aware of hospital-based treatment. Almost all (50; 94.3%) of those affected by leprosy accessed a service at least once a week (either daily or weekly) (Table 5). The three remaining individuals stated that they wished to access the self-care groups more, but did not give a reason why they did not currently attend more often.

### Section 3: Knowledge and perception of the alternate condition and attitudes towards integration

#### Knowledge and relationships with the other participant group

Of the 52 participants affected by LF, 40 (76.9%) were aware of leprosy and 37 (71.2%) stated that they knew someone with the condition (Table 7). The majority of these relationships were slight; of the 37 knowing someone with the condition, 30 (81.1%) categorised the closest relationship they had with a person affected by leprosy as being “someone they saw around”, rather than someone they knew more closely. Only 19 participants affected by LF (47.5% of the 40 participants who were aware of leprosy) believed that it was possible to manage leprosy and its manifestations.

**Table 7:** Awareness and source of information about leprosy by the LF affected group and about LF by the leprosy affected group

Awareness and information source	LF(*n* = 52)	Leprosy (*n* = 53)
Know about the other disease	40	48
Know someone with the other disease	37	43
- Someone they see	30	32
- Someone they know a little	2	2
- A friend	0	5
- A Colleague	2	1
- A Family member	3	3
Know that OWN disease can be managed	48	53
Know that OTHER disease can be managed	19	39

Of the 53 participants affected by leprosy, 48 (90.6%) were aware of LF and 43 (81.0%) stated that they knew someone with the condition. The majority of these 43 participants identified the closest relationship they had with a person affected by LF as being “someone they saw around” (32; 74.4%). A total of 39 participants affected by leprosy (81.3% of the 48 who were aware of the disease) believed that LF could be managed.

The media and Female Community Health Volunteers (FCHVs) were the most frequently reported source of information about the other disease. Of the participants that knew about the other disease, 16 participants affected by LF (40%) and 11 participants affected by leprosy (22.9%) stated the media (radio and/or TV) as their source of knowledge, while 10 participants affected by LF (25.0%) and 14 participants affected by leprosy (29.2%) stated FCHVs as their source (Table 8). Another noteworthy source of information for those affected by leprosy was the SHGs, with 10 participants (20.8%) stating this as their source.

**Table 8:** Source of information about management of the alternate condition

Information source on other disease	Participants that that knew about the other disease		Proportion of participants that did not think that management was possible	
	LF (*n* = 40)	Leprosy (*n* = 48)	LF (*n* = 21)	Leprosy (*n* = 11)
Female Community health volunteer (FCHV)	10	14	60.0%	42.9%
Media	16	11	37.5%	9.1%
SHG	2	10	0.0%	30.0%
Do not know	2	0	100.0%	–
Friends	3	3	100.0%	0%
Health professional	2	3	0.0%	33.3%
Hospital	2	4	50.0%	0.0%
School	0	1	–	0.0%
Person with other disease	2	2	100.0%	0.0%

Regarding the accuracy of information from these sources, 60.0% of participants affected by LF and 42.9% of those affected by leprosy that had obtained information about the other disease from FCHVs did not know that the disease could be managed. Of those that obtained information from the media, 37.5% of participants affected by LF and 9.1% of participants affected by leprosy did not know that the other disease was manageable. Of those that obtained information from the SHGs, all participants affected by LF were aware that leprosy could be managed, and 30% of participants affected by leprosy did not know that LF could be managed.

#### Comparison of participants’ perceptions of leprosy and LF

The mean EMIC score for perception of LF by participants affected by leprosy was 17.9 (95% CI 16.9–19.0), and for leprosy by participants affected by LF was 17.4 (95% CI 16.3–18.5), showing that the perceived stigma between both participants’ groups is not significantly different (*p* = 0.47).

**Fig. 2:**
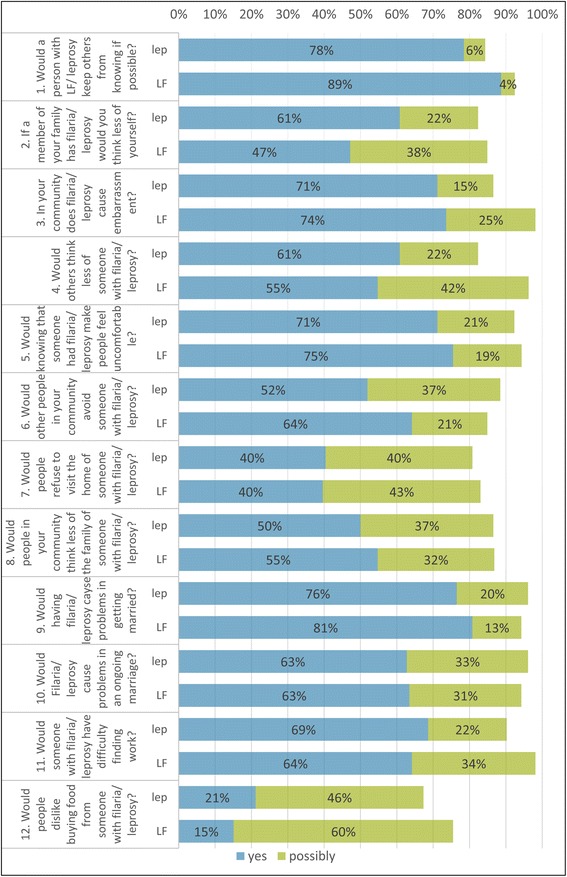
Perception of stigma among participants affected by LF towards leprosy and participants affected by leprosy towards LF. Note: Lep refers to a person affected by leprosy and their perception about LF, and vice versa

Participants responses to the 12 domains of community perceptions of both LF and leprosy are illustrated in Fig. 2. Over 80% of all participants answered ‘yes’ or ‘possibly’ in every domain except the domain regarding buying food from persons with LF or leprosy. When asked, 40 participants affected by LF (78.4%) believed a leprosy-affected person would try to keep their condition secret if possible. Furthermore, 37 participants affected by LF (71.2%) felt that leprosy causes shame and embarrassment, and 31 (60.8%) stated that they would think less of themselves if a member of their family were affected by leprosy. Comparatively, a higher number of participants affected by affected by leprosy believed a person affected by LF would try to keep others from knowing with 47 (88.7%) answering yes and a further two (3.8%) answering possibly. A total of 39 (73.6%) felt that LF causes shame and embarrassment and 25 (47.2%) stated that they would think less of themselves if a member of their family had lymphoedema.

Regarding participants’ perceptions of community attitudes towards the disease, 37 participants affected by LF (71.2%) felt that that knowing someone had leprosy would make others in their community feel uncomfortable, however only 27 (51.9%) thought that people would avoid a person with the disease. Comparatively, 40 participants affected by leprosy (75.5%) claimed that others would feel uncomfortable knowing that a person had LF, while 34 (64.2%) thought that people would avoid them.

The conditions were perceived by both participant groups to affect relationship success, as 39 participants affected by LF (76.5%) believed leprosy would cause difficulty getting married, and 42 participants affected by leprosy (80.8%) believed the same for LF. While 32 participants affected by LF (62.8%) thought that leprosy would create a problem in an established marriage, 33 participants affected by leprosy (63.5%) believed the same for LF.

##### Attitudes towards integrated self-care

**Fig. 3:**
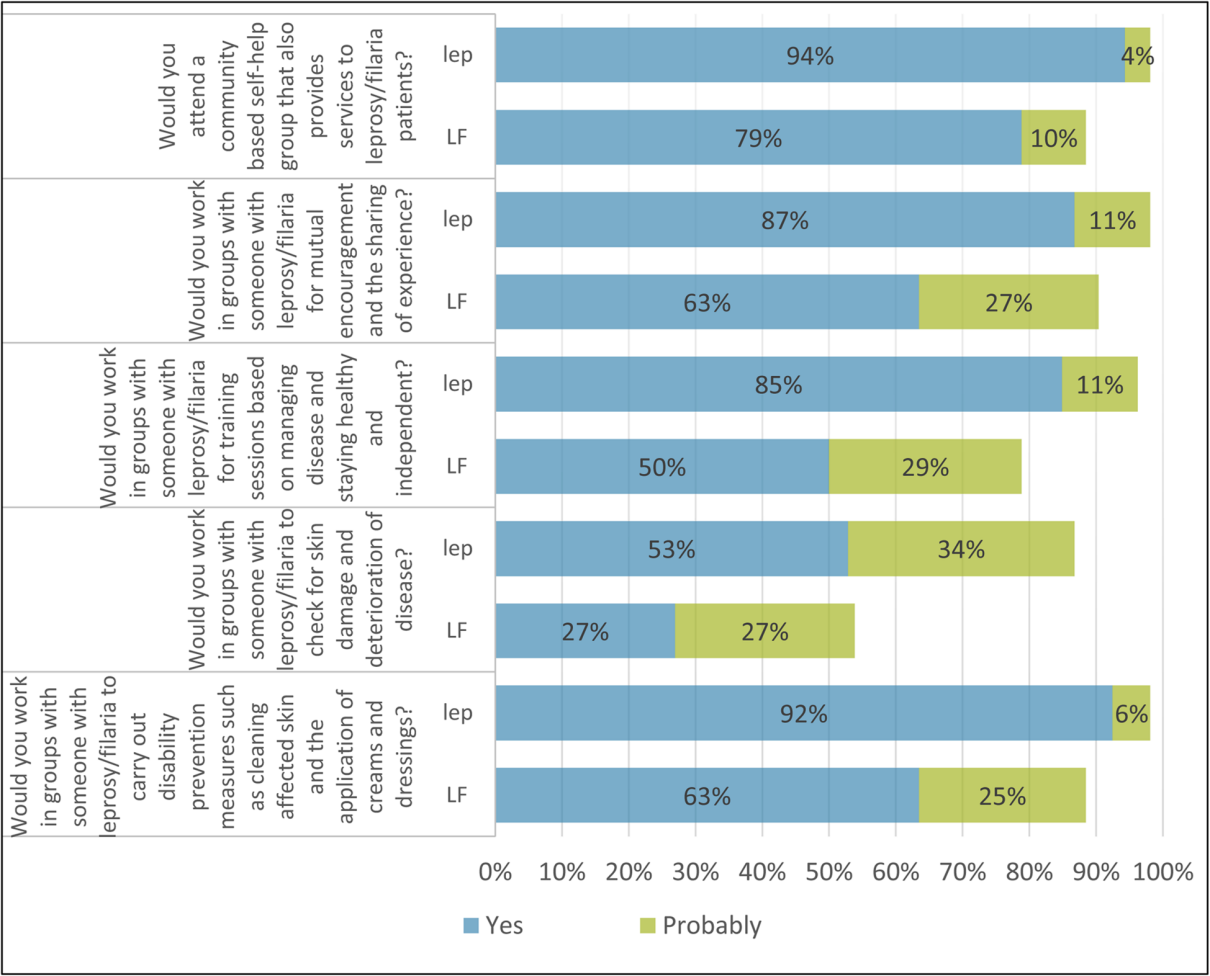
Comparison of attitudes towards integrated self-care for LF and leprosy affected participants in the community. Note: Lep refers to a person affected by leprosy and their perception about LF, and vice versa

In relation to the integration of services, 41 participants affected by LF (78.8%) stated that they would attend a community-based SHG that also provides services to people affected by leprosy, while a further five (9.6%) would probably attend. Leprosy-affected participants were found to be even more willing to integrate, with 50 (94.3%) stating they would attend the group, and an additional two (3.8%) stating that would probably attend. A summary of both participant groups’ willingness to perform various self-care activities in an integrated group is shown in Fig. 3.

## Discussion

While WHO recommends integration for MMDP services for LF and leprosy, this study is the first in Nepal and elsewhere to investigate the feasibility of integrating LF and leprosy care at the community level. Within the two participant groups surveyed, a higher proportion of leprosy-affected participants supported themselves financially, compared to LF-affected participants, 42% of whom relied on family or charitable support. Nearly 77% of the leprosy-affected participants supported themselves with small businesses, growing vegetables or keeping animals. This reflects another feature of the NLT SHGs in that they function as a micro credit unions; members can avail themselves of loans from the group to start up income generating projects. Loans are paid back at interest rates set by the group members. A caveat is that loans can only be applied for after the member has saved a given amount into the group’s funds. Both groups had suffered from the effects of disease for a similar length of time, spanning 10–12 years on average.

The survey revealed that participants affected by leprosy were shown to have significantly greater knowledge of self-care practices than participants affected by LF. Participants affected by leprosy were also implementing such practices more frequently. This result was to be expected given the established involvement of participants affected by leprosy in SHGs, but may be less frequent among those who do not attend a SHG. Despite the relatively low proportion of participants affected by LF practicing self-care, which may be because they are poor and cannot afford to buy soap, cream or shoes, these figures were higher than those in other studies in Nepal, where only a quarter of participants had knowledge of or practiced self-care [27]. Others have reported that self-care is considered to be of particular importance by leprosy SHG members who perceived it to be efficacious for the prevention of worsening disabilities [28].

Whilst the majority of LF-affected participants were aware that some services were available, all participants affected by leprosy were aware that healthcare services were available for their condition (including SHGs). This finding was also to be expected due to the selection of those affected by leprosy through the SHGs. This was reflected in responses regarding the access of participants to services, with only one LF affected participant accessing a service weekly, and 75% never accessing services, compared to 94% of participants affected by leprosy accessing services at least weekly (primarily SHG attendance). Despite the low proportion of participants affected by LF practicing self-care this is higher than other studies in Nepal where only a quarter of participants had knowledge of or practiced self-care [27]. LF affected participants’ limited interaction with health services is consistent with findings from a situational analysis in Ghana where community health workers reported that the management of lymphoedema conditions was rare, and cited the associated social stigma as a key reason for their reclusion [29]. The majority of participants affected by LF stated that they would like to access services more frequently, most commonly SHGs and hospital services, but that cost was the major barrier. This is in line with other studies that have shown that cost of services is a major barrier to patients accessing care [30].

The data showed that those affected by leprosy have greater access to care and greater knowledge of how to manage their condition compared to those affected by LF in the same communities, suggesting that the SHG platform is a good method to increase access and knowledge of self-care for LF affected people in this area. SHG attendance does not have a direct associated cost, though daily wage earners may sacrifice part of their wage to attend a SHG meeting. It is essential that these realities are communicated to people affected by LF to increase access to this service as part of the integrated programme. Additionally, SHGs report that they undertake home visits to ascertain whether people are gaining access to services [21]; this could further increase LF affected peoples’ access to care.

The majority of LF and leprosy participants were aware of the other condition, with greater awareness found by those affected by leprosy. Furthermore, almost half of the participants affected by LF and over 80% of participants affected by leprosy were aware of the other condition knew that it could be managed. The most accurate source of information about the other condition appeared to be the media. Those receiving information from FCHVs appeared to be inaccurately informed as many participants stating FCHVs as their source also believed that the condition could not be managed. While the SHGs were a less frequently stated source of information, this appeared to be an accurate source of information as fewer participants believed that the condition could not be managed. This is concurrent with previous studies in which the SHGs state that raising social awareness of leprosy related issues is an activity readily undertaken by the groups [21], and suggests that increased training and awareness is required for FCHVs in this area.

The EMIC scores for both participant groups were similar, with high scores for leprosy perception by community members reported in other areas [31]. The major aspects of perceived social stigma by both groups were attitudes towards concealment of disease, with over 8O% of participants affected by LF believing people affected by leprosy were likely to keep others from knowing, and over 90% of participants affected by leprosy believing the same for LF. The other domains with high levels of perceived stigma were affected peoples’ embarrassment and community members knowing that someone had the disease would feel uncomfortable. The high EMIC scores may be indicative of high levels of experienced stigma in both groups developed through their own experiences. This is supported by previous reports of people with lymphoedema reporting feelings of isolation and discrimination, including expectations of such behaviour, within their communities [2, 12, 32–36]. In particular, a study of perceptions in Ghana found that while people affected by LF were accepted and cared for by the community, as a result of their own feelings of shame, they did not attend social events [37]. Similarly, studies have shown that people affected by leprosy conceal their condition and withdraw from society before they experienced any negativity in order to maintain social integrity [38, 39]. The extent to which people feel stigmatised, unable to develop good relationships or integrate into communities may also be related to the severity of their condition and the level of disability they experience. This was not examined in the current study, but could provide valuable insights if taken into account in future studies.

Many individuals in both groups felt that the other condition would cause problems in ongoing marriages, as well as issues in individuals with the other condition getting married. Again, this is supported by previous studies examining the marital difficulties, worries and experiences of people affected by lymphoedema [2, 12, 33–35, 37, 40] and people affected by leprosy [14, 41, 42]. This supports the assumption that the high EMIC scores are in part due to experiences of the participants due to their own condition and perceived and/or internalised stigma.

Despite the high levels of perceived stigma, attitudes towards integration of services were positive with more than 85% of the LF affected participants stating that they would attend, or were likely to attend such an integrated SHG. There are multiple elements that likely contribute to this enthusiasm which included the following

- lymphoedema impacts significantly on almost all aspects of an affected person’s life, affecting their economic productivity and capacity to participate socially [3]
- recurring episodes of acute dermatolymphangioadenitis (ADLA) cause additional pain and disability, and depressive disorders are also widespread [43]
- existing care services have been shown by this study to be costly and inaccessible, while the local knowledge of self-care strategies is poor.
- people affected by LF are in desperate need of MMDP services and have limited knowledge of where to turn, and of the means to access such services.

Participants affected by leprosy that already access the SHGs were shown to be even more willing to integrate, with a higher proportion of the participants affected by leprosy willing to perform all activities when compared to participant affected by LF. This finding reflects the eagerness of SHG members to help others in their community, previously identified as a key motivation behind SHG facilitation [21]. The higher proportion of participants affected by leprosy willing to perform each of the activities may also be explained by the existing membership of groups performing such activities, and therefore a greater understanding of what this entails. LF participants’ willingness may change following training and opportunity to integrate into the SHGs.

Previous studies have shown that the SHGs in this area have a significant impact at the community level, increase participation and improve perceptions in the local community of and increase community trust [20, 21, 28]. This, coupled with the enthusiasm of the participants affected by LF to join the SHGs, indicates that integration of people affected by LF into the groups would be a welcome and positive step towards increasing LF affected peoples’ access to care.

## Conclusion

This study concludes that the integration with community-based leprosy SHG services could increase LF affected peoples’ knowledge and practice of self-care. The findings suggest that expansion of the SHGs to deliver MMDP services to LF affected people is feasible in terms of the willingness of both existing and prospective members. From a wider perspective, the integration of MMDP programmes presents an opportunity to meet the complex needs of global NTD control. While significant progress has been made in recent decades, achieving ambitious disease elimination targets remains challenging. The strain on resources can be ameliorated through co-ordination of activities of partner organisations and government sectors sharing a commitment to NTD control to contribute to the scale-up of NTD programmes that is required to achieve elimination goals.

## Abbreviations

ADLA: Acute dermatolymphangioadenitis
CDD: Community development department
FCHV: Female community health volunteers
LF: Lymphatic filariasis
LLHSC: Lalgadh leprosy hospital and services centre
MDT: Multi-drug therapy
MMDP: Morbidity management and disability prevention
NLT: Nepal leprosy trust
NTD: Neglected tropical diseases
ODK: Open data kit
SD: Standard deviation
SHG: Self-help group
VDC: Village development committee
